# Epigenetic modifications of the immune-checkpoint genes *CTLA4* and *PDCD1* in non-small cell lung cancer results in increased expression

**DOI:** 10.1186/s13148-017-0354-2

**Published:** 2017-05-11

**Authors:** Sebastian Marwitz, Swetlana Scheufele, Sven Perner, Martin Reck, Ole Ammerpohl, Torsten Goldmann

**Affiliations:** 1 Campus Luebeck and the Research Center Borstel, Leibniz Center for Medicine and Biosciences, Pathology of the University Medical Center Schleswig-Holstein, 23538 Luebeck, Germany; 2 Institute of Human Genetics, Christian-Albrechts-University Kiel and University Medical Center Schleswig-Holstein, Campus Kiel, Kiel, Germany; 3 LungenClinic Großhansdorf, Großhansdorf, Germany; 4 Airway Research Center North (ARCN), Member of the German Center for Lung Research (DZL), Großhansdorf, Germany

**Keywords:** PD-1, PD-L1, Methylome, Immune-checkpoint, NSCLC

## Abstract

Targeting checkpoint inhibitors using monoclonal antibodies results in significantly better outcome of cancer patients compared to conventional chemotherapy. However, the current companion diagnostics to predict response is so far suboptimal, since they base on more or less reliable immunohistochemical approaches. In order to overcome these limitations, we analyzed epigenetic modifications of *PDCD1* (PD1), *CD274* (PD-L1), and *CTLA4* in NSCLC tissues from 39 patients. Results were correlated with transcriptome data. Significant differences in the CpG-methylation patterns between tumor tissues and matched controls were observed for *CTLA4* and *PDCD1* (PD1) showing a decreased methylation of these genes compared to matched tumor-free tissues from the same patients. Results were confirmed by bisulfide sequencing in an independent validation cohort. Hypomethylation also resulted in increased expression of these genes as shown by transcriptome data. These epigenetic pathways as a hallmark of NSCLC might be useful to generate more precise diagnostic approaches in the future.

## Main text

In contrast to identification of well-defined oncogenic alterations like EGFR mutations for patient stratification, effective selection of predictive biomarkers remains a challenge in the era of checkpoint blockade. Recently, PD-L1 copy number gain has been reported for a subset of non-small cell lung cancer (NSCLC) [1] indicating comparably rude genetic changes taking place. In order to gain a more precise impression of the biology of these molecules, we analyzed the presence of epigenetic modifications and RNA-transcription of *PDCD1* (PD1), *CD274* (PD-L1), and *CTLA4* in a set of patient tissues.

NSCLC and matched tumor-free lung tissues were obtained from patients who underwent surgery with curative intend at the LungenClinic Großhansdorf. The use of patient material was approved by the local ethics council at the University of Lübeck (AZ-12-220). A total of 39 patients were included for array-based methylation analyses. From 18 of these patients, RNA was extracted for transcriptome analysis. An independent validation cohort of 20 patients was used for bisulfite sequencing. For all analyses, H&E stained tissue specimen were examined under a microscope and tumor cells were microdissected to obtain material of high purity for downstream RNA/DNA analysis.

To investigate epigenetic modifications in tumor tissues and matched controls, DNA methylation analysis of HOPE-fixed tissues using HumanMethylation450 BeadChips was performed as described in detail elsewhere [2]. Bisulfite pyrosequencing of the following loci was performed for validation purposes as described before [3]: cg25798782 (chr2: 242,795,283; forward primer: ttagggagatttaagttagagttag; reverse primer (biotinylated): accacctactcacatccct; sequencing primer: tgtagtggaggttagt), cg08460026 (chr2: 204,732,475; forward primer: atgtgtatatatagaaggtatttgaatag; reverse primer (biotinylated): aatctccacttaattatccaaatcct; sequencing primer: tagaaggtatttgaatagaa) and cg26091609 (chr2: 204,734,182; forward primer: ttgtgttgtatgatgttatttatttgttt; reverse primer (biotinylated): actataatctaactaactaaaactactaa; sequencing primer:tttatattagagatattagttt).

Transcriptome analysis from human tissue samples was conducted as described elsewhere [4]. Quantile-normalized relative gene expression values for *CTLA4* (NM_005214), *PDCD1* (PD1) (NM_005018), and *CD274* (PD-L1) (NM_014143) were obtained from GEO dataset GSE74706 and analyzed with GraphPad Prism v.7.

**Fig. 1:**
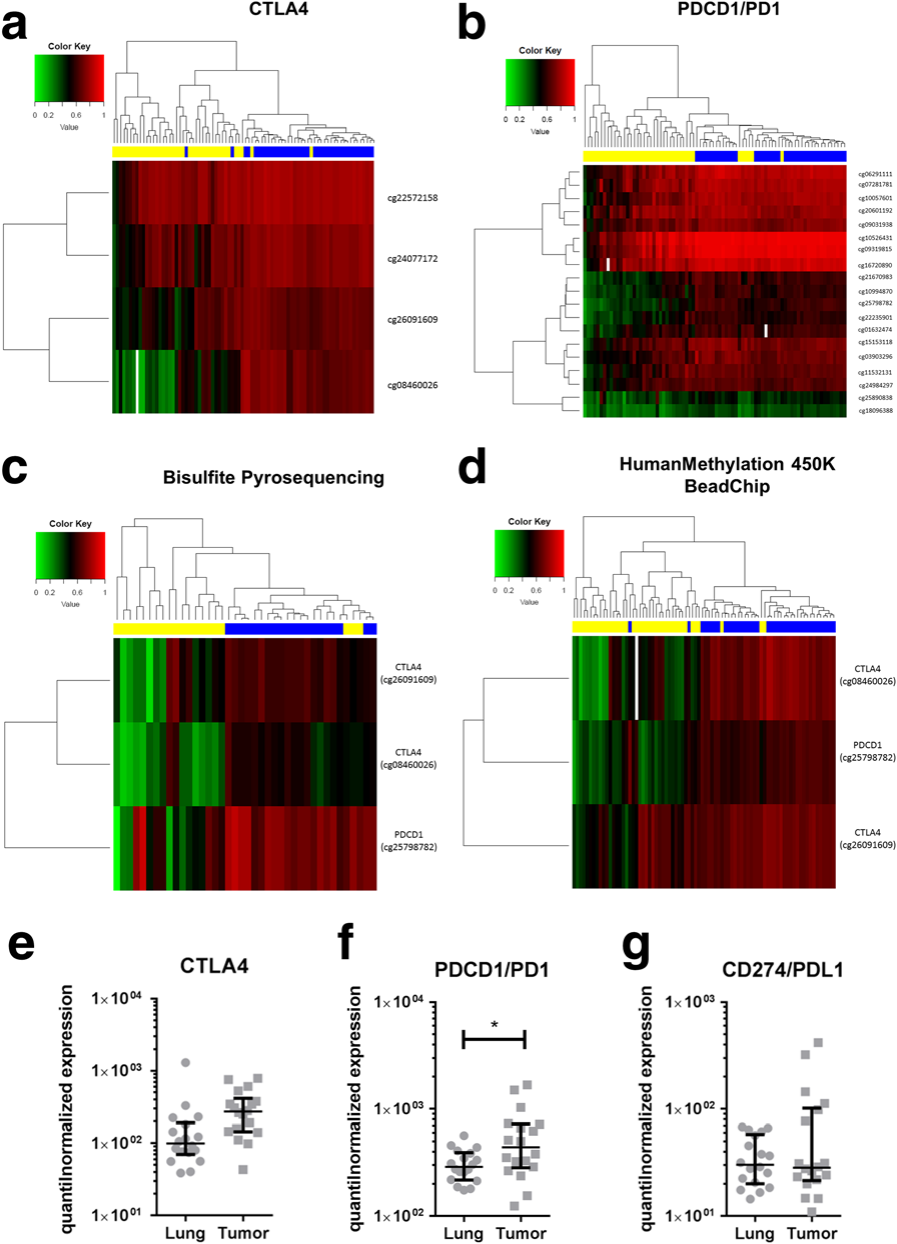
Epigenetic and gene expression analyses of immune checkpoint molecules in human NSCLC and corresponding control tissues. HumanMethylation450k BeadChip data, obtained from 39 tumor tissues and their corresponding controls, identified multiple CpG loci differentially methylated in CTLA4 **a** and PDCD1 (PD1) **b** genes (FDR < 0.01, *t* test). For data validation bisulfite pyrosequencing **c** of an independent patient cohort (*n* = 20) was performed, confirming differential methylation of tumor tissues detected previously via HumanMethylation450k BeadChip **d** at selected loci (CTLA4: cg08460026 and cg26091609, PDCD1 (PD1): cg25798782). Heatmap.2 was utilized for hierarchical cluster analysis and data visualization. *Blue bars* on top of heatmaps: tumor-free lung tissue, *yellow bars*: tumor tissue. Transcriptome analyses of 18 patients showed elevated transcript expression of *CTLA4* **e** *PDCD1* (*PD1*) Transcriptome analyses of 18 patients showed elevated transcript expression of CTLA4 e PDCD1 (PD1) **f** and, to a lesser degree, CD274 (PD-L1) **g** in the tumor samples. Transcriptome data was analyzed via paired *t* test of quantile-normalized, relative gene expression values with *p* ≤ 0.05 (=*) regarded as significant

Using array-based analyses, significant differences in the CpG-methylation patterns between tumor tissues and matched controls were observed for *CTLA4* and *PDCD1* (*PD1*) (FDR < 0.01; Fig. 1a, b): NSCLC tumors exhibited a decreased degree of CpG-methylation in these loci compared to tumor-free tissues. No significant differences in methylation pattern for *CD274* (*PD-L1*) could be observed (data not shown). Due to these findings, several CpG loci located in *CTLA4* and *PDCD1* (*PD1*) were selected for validation via bisulfite pyrosequencing. Bisulfite pyrosequencing verified the results obtained by array-based analysis (Fig. 1c). These findings point towards reduced methylation levels in CpGs islands of the immune-checkpoint molecules *CTLA4* and *PDCD1* (*PD1*), which might positively influence gene expression. Our findings from DNA level were found to be further reflected on gene expression level: elevated mRNA-transcription in the tumors of the same patients (Fig. 1e–g) were detected and in case of *PDCD1* (*PD1*) (Fig. 1f) significantly upregulated. Our data shows for the first time, that *CTLA4* and *PDCD1* (*PD1*) are epigenetically modified in human lung tumors, which is furthermore associated with increased transcription. Together with the copy number gains observed for *CD274* (*PD-L1*), these epigenetic pathways as a hallmark of NSCLC might be used for more precise diagnostic approaches in the future. Recent data proposes that modulation of DNA methylation via methyltransferase inhibitors might trigger anti-tumor immune responses [5]. The data presented here indicates already hypomethylated *PDCD1* (PD1) and CTLA4 CpGs in the tumor cells, which could be of influence when addressing such kind of treatments. As a matter of future studies, the potential differences in circulating tumor as well as immune cells compared to the tissue infiltrating immune cells or the resident tumor cells should be analyzed. Furthermore, using epigenetic analyses as well as transcriptomic approaches to investigate the underlying complexity of a disease with respect to current therapeutic regimens, will create links between classical molecular pathology with epidemiology(MPE) and will enable holistic studies as discussed elsewhere [6].

## Abbreviations

NSCLC: Non-small cell lung cancer
